# Neuropsychiatric Symptoms Mediated the Relationship Between Odor Identification and Cognition in Alzheimer's Disease Spectrum: A Structural Equation Model Analysis

**DOI:** 10.3389/fnagi.2021.732840

**Published:** 2022-01-12

**Authors:** Qiang Wang, Ben Chen, Xiaomei Zhong, Huarong Zhou, Min Zhang, Naikeng Mai, Zhangying Wu, Xinru Chen, Mingfeng Yang, Si Zhang, Gaohong lin, Thomas Hummel, Yuping Ning

**Affiliations:** ^1^Department of Geriatric Psychiatry, Memory Clinic, The Affiliated Brain Hospital of Guangzhou Medical University, Guangzhou Huiai Hospital, Guangzhou, China; ^2^Department of Geriatric Psychiatry, The Second People's Hospital of Dali Bai Autonomous Prefecture, Dali, China; ^3^Department of Neurology, The Affiliated Brain Hospital of Guangzhou Medical University, Guangzhou Huiai Hospital, Guangzhou, China; ^4^Department of Otorhinolaryngology, Smell and Taste Clinic, Technische Universität Dresden, Dresden, Germany; ^5^The First School of Clinical Medicine, Southern Medical University, Guangzhou, China; ^6^Guangdong Engineering Technology Research Center for Translational Medicine of Mental Disorders, Guangzhou, China

**Keywords:** neuropsychiatric symptoms, odor identification, cognition, Alzheimer's disease, mild cognitive impairment, structural equation modeling

## Abstract

**Background:** Odor identification dysfunction is an early predictor of the development of Alzheimer's disease (AD), but neuropsychiatric symptoms (NPS), which are common in AD and mild cognitive impairment (MCI), are also associated with odor identification dysfunction. Whether NPS affect the specificity of using odor identification dysfunction to predict cognitive decline in AD and MCI remains unclear.

**Methods:** Patients (233 with MCI and 45 with AD) and 45 healthy controls (HCs) underwent assessments of odor identification (Sniffin' Sticks), NPS (Neuropsychiatric Inventory-12), and cognitive function (global cognition, memory, language, executive function, visual-spatial skill, and attention). Structural equation modeling (SEM) with bootstrapping estimation was conducted to explore the relationships between odor identification, NPS, and cognition.

**Results:** Patients with NPS showed significantly worse performance in odor identification and cognition than patients without NPS and HCs. The SEM showed odor identification to be positively associated with cognition, and cognition had special indirect effects on odor identification through affective and psychosis symptoms (two factors extracted from Neuropsychiatric Inventory-12). Additionally, affective and psychosis symptoms partially mediated the effect of cognition on odor identification.

**Conclusion:** Neuropsychiatric symptoms are associated with odor identification dysfunction in patients with AD and MCI. Studies exploring the relationship between odor identification dysfunction and cognitive decline in patients with AD and MCI should include an assessment of affective and psychosis symptoms, and adjust their confounding effects.

## Background

Odor identification dysfunction is commonly observed in Alzheimer's disease (AD) (85–90%) (Woodward et al., 2017) and mild cognitive impairment (MCI) (47–65%) (Velayudhan, 2015; Wang et al., 2021). It precedes cognitive decline and clinical manifestations of AD and MCI and may be paralleled with tau-mediated neuronal damage during disease progression (Murphy, 2019). Poor odor identification has been repeatedly shown to be associated with worse general cognitive performance (Wang et al., 2021), increased cortical amyloid burden (Bahar-Fuchs et al., 2010), and lower ratios of CSF t-tau and P_181_-tau to Aβ_1−42_ (Lafaille-Magnan et al., 2017). Additionally, longitudinal studies suggested that poor odor identification predicts cognitive decline and conversion to major neurocognitive disorder in amnestic MCI subjects (Devanand et al., 2015; Roberts et al., 2016) and in older individuals with normal cognitive function (Djordjevic et al., 2008). A recent study also showed a combination of Aβ_1−42_ and odor identification scores to improve the predictive accuracy of conversion from MCI to AD (Zhao et al., 2020). These observations suggest that odor identification dysfunction serves as a non-invasive and cost-effective marker for predicting cognitive decline in AD spectrum disease.

Numerous studies have found a close relationship between odor identification dysfunction and neuropsychiatric symptoms (NPS) such as depression, anxiety, and psychosis symptoms (Ropacki and Jeste, 2005; Moberg et al., 2014; Croy and Hummel, 2017; Kamath et al., 2018). With regard to affective symptoms, patients with olfactory loss are more likely to exhibit symptoms of depression and anxiety (Croy et al., 2010), and patients with major depression exhibit impaired odor identification (Chen et al., 2019). In addition, olfactory dysfunction recovers with the remission of depressive symptoms (Zucco and Bollini, 2011). In patients with late-life depression, those with odor identification dysfunction exhibit poorer cognitive performance and more structural and functional brain abnormalities (Chen et al., 2018, 2021). Likewise, high-trait anxiety individuals detect odors faster than low-trait anxiety participants, and trait anxiety levels are negatively correlated with the speed of reactions to odors (La Buissonnière-Ariza et al., 2013). With respect to psychosis symptoms, a meta-analysis found moderate to high olfactory dysfunction in schizophrenia patients (Moberg et al., 2014), and poorer odor identification scores were found to be associated with longer disease duration (Moberg et al., 2006). Moreover, typical characteristics of schizophrenia, such as negative symptoms and lower intelligence, were found to be related to odor identification dysfunction (Corcoran et al., 2005), and odor identification dysfunction is also reported in first-degree relatives and monozygotic twins of schizophrenia patients (Ugur et al., 2005; Turetsky et al., 2008). Overall, these findings show that odor identification dysfunction is strongly associated with NPS, especially for affective and psychosis symptoms.

Notably, various kinds of NPS are common manifestations in patients with AD and MCI, including depression, anxiety, apathy, delusions, hallucinations, episodes of verbal, and physical aggression, etc. (Ropacki and Jeste, 2005; Hollingworth et al., 2006). The prevalence of NPS in patients with AD is 56–98% in the community and up to 91–96% in hospitals and long-term care facilities (Gerlach and Kales, 2018). For MCI, NPS have been reported in 35–85% of patients and may occur prior to cognitive decline (Gallagher et al., 2017). Additionally, NPS are associated with greater functional impairment, poorer quality of life, accelerated cognitive decline and a more significant degree of AD neurodegeneration (Kales et al., 2015).

All of these studies illustrate that odor identification dysfunction, cognitive decline, and NPS are closely intertwined, raising the question of whether the prediction of cognitive decline by odor identification dysfunction may be affected by NPS in patients with AD and MCI. Considering what is mentioned above, we hypothesized that the relationship between odor identification dysfunction and cognitive decline is mediated by NPS (especially affective and psychosis symptoms) in patients with AD and MCI. The present study aimed to explore the relationship between NPS, odor identification dysfunction, and cognitive decline in patients with AD and MCI via structural equation modeling (SEM) analysis. The results provide a deeper understanding of how odor identification dysfunction, cognitive decline, and NPS interact with each other and contribute to the rational use of odor identification in clinical practice.

## Materials and Methods

### Participants

In total, 233 subjects with MCI and 45 with AD were continuously recruited from the Affiliated Brain Hospital of Guangzhou Medical University (Guangzhou Huiai Hospital), and 45 age-matched healthy controls (HCs) were recruited from communities in Guangzhou. All subjects or their legal guardians provided signed informed consent to participate in the study. The present study was approved by the Ethics Committees of the Affiliated Brain Hospital of Guangzhou Medical University (Guangzhou Huiai Hospital). All procedures performed in this study were done in accordance with the 1964 Helsinki declaration and its later amendments or comparable ethical standards.

The diagnosis of probable AD was defined according to the clinical criteria of the National Institute of Neurological and Communicative Disorders and Stroke-Alzheimer's Disease and Related Disorders Association (Mckhann et al., 1984), and diagnostic criteria for MCI were based on the Peterson criteria (Petersen, 2004). All recruited subjects with a Hachinski score of higher than four were also excluded (Hachinski et al., 1975). The other exclusion criteria were as follows. (1) Major systemic, past, or concomitant diagnoses of psychiatric disorders (such as major depression, schizophrenia, bipolar disorder, posttraumatic stress disorders, panic disorder, etc.); (2) patients with a history of concomitant diagnosis of any neurodegenerative disease aside from AD; (3) other causes (infectious, toxic, and metabolic) of cognitive impairment were excluded; (4) Other causes that significantly influence olfaction, including active upper respiratory/sinus infection or respiratory distress at the time of testing, congenital or traumatic anosmia, known nasal polyps or tumors, current or recent (past 6 months) smoking, and alcohol or substance dependence were also excluded. In addition, a trained psychologist, via dedicated clinical interviews, carefully screened all HCs to exclude any evidence of psychopathological symptoms. All subjects completed structured interviews, standardized olfactory tests, and clinical symptom and comprehensive cognitive assessments on the same day.

### Assessments

#### Assessments of Odor Identification

Odor identification function was assessed using the standardized Sniffin' Sticks Screen 16 test (Hummel et al., 1997) which involves the presentation of odorants through felt-tip pens. For odor performance, the cap of a pen was removed, and the pen's tip was placed approximately 2 cm in front of the participant's nostrils for 3 s. Subjects were presented with 16 common odorants. Odors were identified from flash cards listing four verbal odor descriptors each (forced choice, score range of 0–16). Olfactory testing was performed in a quiet, odorless, and well-ventilated room at the Affiliated Brain Hospital of Guangzhou Medical University.

#### Assessments of Cognitive Function

Cognitive function in different domains was evaluated by the following neuropsychological tests: the mini mental state examination (MMSE) (Folstein et al., 1975), auditory verbal learning task (AVLT) (Zhao et al., 2012), trail-making test (TMT) (Lu et al., 2006), symbol-digit modality test (SDMT) (Sheridan et al., 2006), boston naming test (BNT) (Guo et al., 2006), and ReyO-sterrieth complex figure (ROCF) test (Guo et al., 2000). The scores of the MMSE represent global cognition. The time take to complete TMT Part B was used to represent executive function. The AVLT N1-3, BNT, ROCF, and SMDT scores represent memory, language, visual-spatial skill, and attention, respectively.

#### Assessments of Neuropsychiatric Symptoms

Neuropsychiatric symptoms were measured using the Chinese Neuropsychiatric Inventory (NPI) originally proposed by Cummings et al. (1994) and Kaufer et al. (1998). Neuropsychiatric symptoms were scored by monitoring caregivers' responses obtained from a self-reporting questionnaire wherein they selected the frequency (four-point scale) and severity (three-point scale) of symptoms. The frequency and severity scores for each symptom were multiplied as symptom scores with a higher symptom score indicating higher severity (including delusions, hallucinations, agitation, irritability, depression, anxiety, apathy, euphoria, disinhibition, aberrant motor behavior, and sleep and eating disorders). The sum of 12 kinds of symptom scores was defined as the NPI total score.

Patients with NPI total scores of ≥1 were defined as the NPS group, and those with total scores of = 0 were defined as other patients classified as the No-NPS group.

### Statistical Analysis

The data were analyzed using the Statistical Package for the Social Sciences version 26.0 (SPSS 26.0) and Amos 24.0 programs (https://www.ibm.com/products/spss-statistics). Neuropsychiatric symptoms were classified by using factor analysis. Before performing factor analysis, the suitability of the data was tested using the Kaiser–Meyer–Olkin (KMO) and Bartlett tests. The internal reliability of the classified NPS symptoms was measured using Cronbach's α. Exploratory factor analysis (EFA) was performed using maximum likelihood analysis followed by varimax factor rotation. Based on the EFA results, models of factorial grouping for the NPI-12 were established using confirmatory factor analysis (CFA). The correlation between the measured variables were determined using the partial correlation coefficient, and age, sex, and years of education were set as control variables.

Structural equation modeling was used to test the hypothesized model. According to Jöreskog and Sörbom (1982), SEM provides a maximum-likelihood estimation of the entire system of a hypothesized model and enables the assessment of variables with data. The error variance of a single variable is determined by the following formula: error variance of X1 = (1 − reliability coefficient) ^*^ (*S*^2^) (Randall and Schumacker, 2010). In the present analysis, we adopted Anderson and Gerbing (1988) two-step strategy to test the hypothesized model. First, the measurement model was confirmed using CFA, and then we performed a SEM analysis to measure the fit and path coefficients of the hypothesized model. The chi-square (χ^2^) value, degrees of freedom (*df* ), the value of χ^2^/*df* , the goodness of fit (GFI), the comparative fix index (CFI), Bollen's incremental fit index (IFI), the normed fit index (NFI), the standardized root mean square residual (SRMR), and the root mean square error of approximation (RMSEA) were adopted to estimate model fit. The significance of the effects of the study model was tested using the bootstrapping method (10,000) (Hayes, 2009).

## Results

### Demographic, Olfactory, and Cognitive Information

Demographic, olfactory, and cognitive information is shown in Table 1. In total, 168 patients were grouped into the NPS group, and 110 were grouped into the No-NPS group.

**Table 1:** Demographic, olfactory, and cognitive information of NPS and No-NPS patients.

**Whole sample**	**NPS**	**No-NPS**	**HCs**	**^#^*F*/χ^2^**	***p***	***Post-hoc^a^***
	***n* = 168**	***n* = 110**	***n* = 45**			
**Age** (years)	67.3 ± 8.8	69.1 ± 9.5	65.6 ± 8.8	2.866	0.058	–
**Education** (years)	8.9 ± 3.4	10.2 ± 3.4	11.4 ± 2.9	12.587	<0.01^**^	A < B < C
**Sex** (male/female)	53/115	34/76	15/30	0.087	0.957	–
**MMSE**	22.3 ± 5.3	24.9 ± 3.6	27.0 ± 2.2	24.970	<0.01^**^	A < B < C
**Odor identification**	9.3 ± 2.7	10.5 ± 2.3	12.1 ± 1.6	15.576	<0.01^**^	A < B < C
**AD/MCI**	38/130	7/103	–	–	–	–
**Memory**						
Auditory verbal learning N1–3	9.6 ± 4.8	12.2 ± 4.6	14.4 ± 3.1	20.551	<0.01^**^	A < B < C
**Language**						
Boston naming test	18.9 ± 3.8	20.0 ± 3.5	23.3 ± 2.4	29.784	<0.01^**^	A < B < C
**Executive function**						
Trail-making test	85.9 ± 33.7	79.3 ± 34.9	55.9 ± 17.9	14.472	<0.01^**^	A >B > C
**Visual-spatial skill**						
Rey's complex figure copy	22.9 ± 6.4	24.4 ± 6.2	28.4 ± 3.3	14.794	<0.01^**^	A < B < C
**Attention**						
Symbol-digit modality test	27.1 ± 9.7	30.9 ± 10.9	36.3 ± 9.8	15.977	<0.01^**^	A < B < C

*AD, Alzheimer's disease; MCI, mild cognitive impairment; NPS, neuropsychiatric symptoms; no-NPS, no neuropsychiatric symptoms; HC, healthy control; MMSE, mini mental state examination*.

# *F refers to the two-tailed Fisher's exact test (two-tailed), χ^2^ refers to the two-tailed chi-square test, and*

** *p < 0.01*.

a *In post-hoc multiple comparisons, A denotes the NPS group, B denotes the No-NPS group, and C denotes the HCs group*.

### Exploratory Factor Analysis of Neuropsychiatric Symptom Clusters

**Figure 1:**
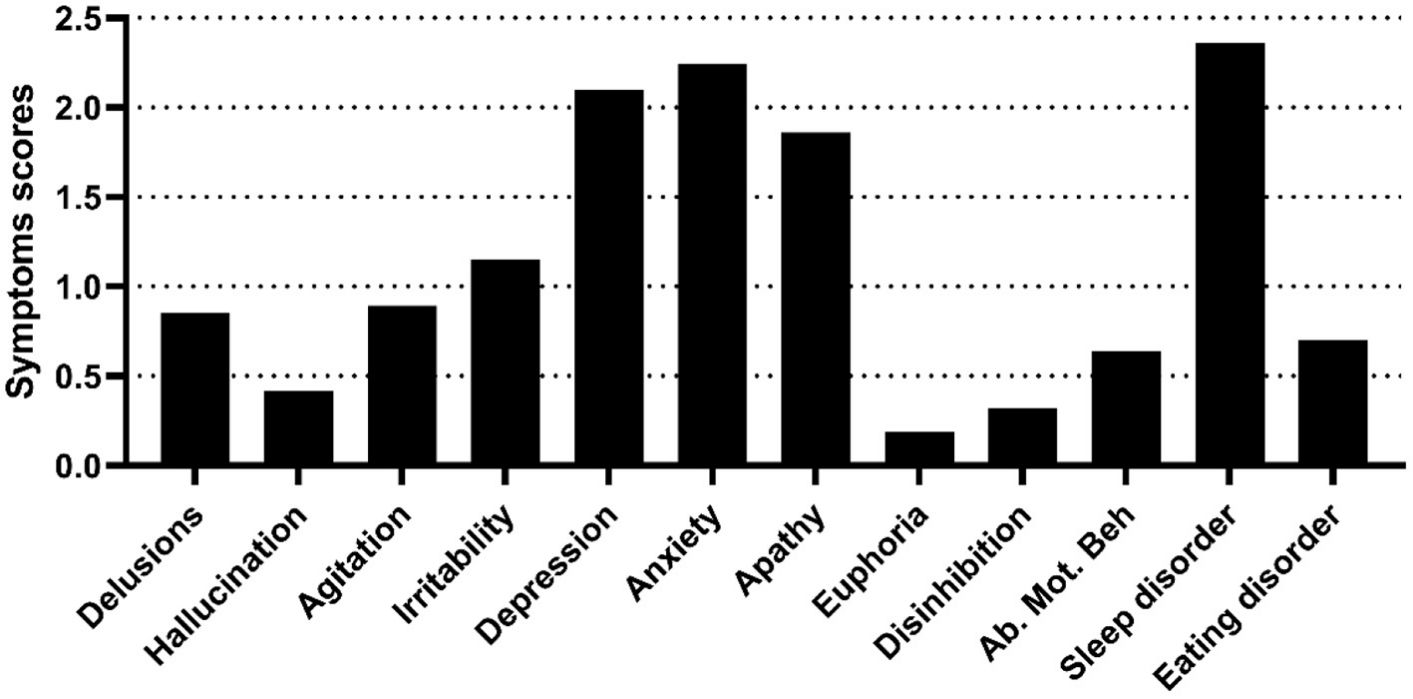
Neuropsychiatric symptoms of the NPS group. The figure shows the symptom scores of the NPS group, as assessed by the NPI-12. Ab. Mot. Beh, aberrant motor behavior.

The scores for each symptom as shown in Figure 1. Based on the symptom scores for NPS, three factors were extracted via the EFA. The variances for Factor 1, Factor 2, and Factor 3 were 2.27, 2.23, and 1.85, respectively. The explanatory power values of Factor 1, Factor 2, and Factor 3 were 18.9, 18.5, and 17.9%, respectively. The total explanatory power of the three factors represented 55.4% of the total variance.

Symptom Cluster 1 (Factor 1) described affective symptoms, including depression, anxiety and apathy. Symptom Cluster 2 (Factor 2) described psychosis symptoms, including delusions, hallucinations, agitation, and irritability. Symptom Cluster 3 (Factor 3) described behavioral symptoms, including disinhibition, euphoria, aberrant motor behavior, and eating and sleep disorders (Table 2).

**Table 2:** Communalities and rotated factor matrix of the NPS group.

**Neuropsychiatric symptoms**	**Initial**	**Extraction**	**Factor 1**	**Factor 2**	**Factor 3**
Anxiety	0.691	0.783	0.857	0.163	0.151
Depression	0.628	0.706	0.835	0.012	0.092
Apathy	0.691	0.783	0.828	0.273	0.125
Delusions	0.639	0.765	0.084	0.864	0.103
Hallucination	0.551	0.436	0.057	0.656	0.049
Agitation	0.629	0.406	0.098	0.628	0.045
Irritability	0.551	0.332	0.105	0.563	0.057
Disinhibition	0.721	0.876	0.033	0.062	0.933
Euphoria	0.737	0.78	0.008	0.338	0.816
Ab. Mot. Beh	0.527	0.447	0.237	0.332	0.53
Eating disorder	0.294	0.191	0.077	−0.077	0.423
Sleep disorder	0.219	0.146	0.235	0.006	0.301

*Extraction method: maximum likelihood. Rotation method: Varimax with Kaiser normalization. Rotation converged in five iterations. Ab. Mot. Beh, aberrant motor behavior*.

The KMO measure for this study was measured as 0.681, indicating an appropriate sample size. In addition, Bartlett's test of sphericity showed that statistical significance was <0.001, thereby confirming the goodness-of-fit of the model.

### Confirmatory Factor Analysis of Neuropsychiatric Symptom Clusters

**Figure 2:**
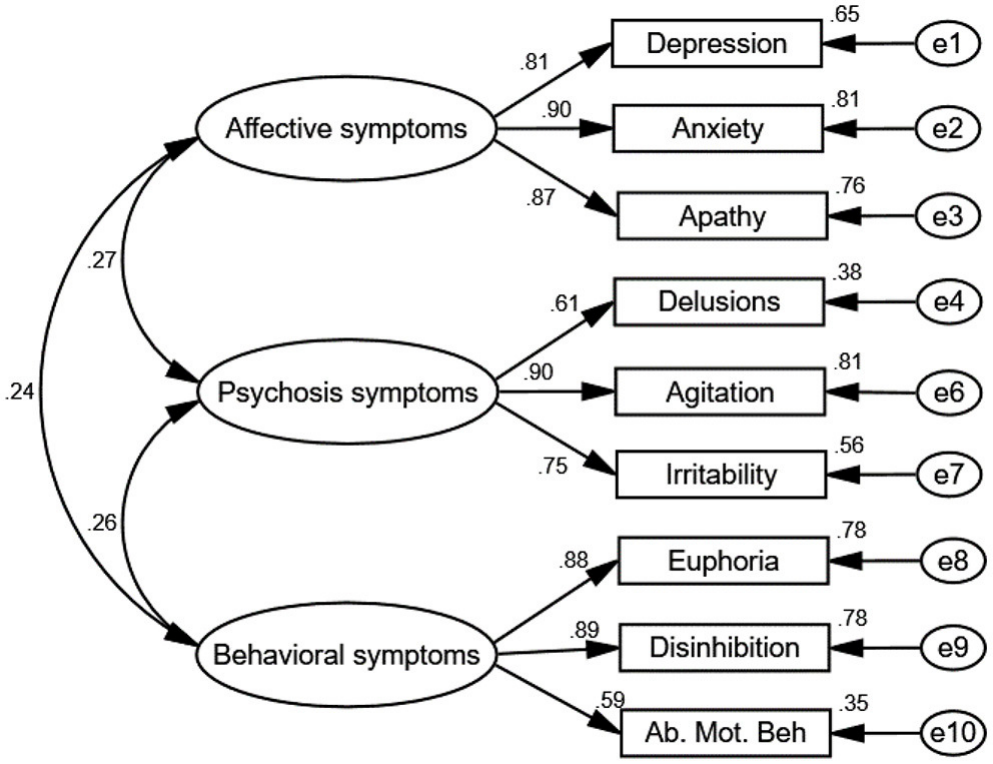
Path diagram of the confirmatory factor analysis. The goodness-of-fit indices of the modified model were as follows: χ^2^/*df* = 2.06, GFI = 0.96, CFI = 0.98, NFI = 0.96, IFI = 0.98, and RMSEA = 0.06, which indicate a good fit. Three factors were verified from the CFA: Factor 1 described affective symptoms, including depression, anxiety, and apathy. Factor 2 described psychosis symptoms, including delusions, agitation, and irritability. Factor 3 described behavioral symptoms, including disinhibition, euphoria, snf aberrant motor behavior. Rotation converged in nine iterations. Ab. Mot. Beh, aberrant motor behavior. e1–10 represent residuals of the respective variables.

The results of testing the goodness of fit of the CFA show that GFI = 0.786, CFI =0.809, IFI = 0.789, χ^2^/*df* = 5.096, and RMSEA = 0.157, indicating a poor fit. To improve reliability, the model was modified by removing variables with factor loadings of <0.6 (hallucinations and sleep and eating disorders) (Bagozzi and Yi, 1989) while simultaneously verifying the model fit (Figure 2). Successively, the goodness-of-fit indices of the modified model were χ^2^/*df* = 2.06, GFI = 0.96, CFI = 0.98, NFI = 0.96, IFI = 0.98, and RMSEA = 0.06, indicating a good fit.

### Correlation Between Clinical Characteristics and Odor Identification in Subjects With NPS

The odor identification score was positively correlated with cognitive scores, and negatively correlated with scores of psychosis and affective symptoms in the NPS group (*P* < 0.05). There was no significant correlation between odor identification and behavioral symptoms (Table 3).

**Table 3:** Correlation between cognition, NPS, and odor identification in patients with NPS (*N* = 168).

**Symptoms**	**1**	**2**	**3**	**4**	**5**	**6**	**7**	**8**	**9**	**10**
Odor identification	1.00									
Global cognition	0.43^**^	1.00								
Memory	0.44^**^	0.62^**^	1.00							
Executive function	−0.32^**^	−0.61^**^	−0.52^**^	1.00						
Language	0.36^**^	0.52^**^	0.51^**^	−0.43^**^	1.00					
Visual-spatial skill	0.29^**^	0.57^**^	0.42^**^	−0.44^**^	0.44^**^	1.00				
Attention	0.26^**^	0.48^**^	0.50^**^	−0.52^**^	0.38^**^	0.37^**^	1.00			
Affective symptoms	−0.32^**^	−0.29^**^	−0.23^**^	0.22^**^	−0.22^*^	−0.19	−0.09	1.00		
Psychosis symptoms	−0.39^**^	−0.45^**^	−0.38^**^	0.48^**^	−0.31^**^	−0.20^*^	−0.39^**^	0.04	1.00	
Behavioral symptoms	−0.15	−0.14	−0.19^*^	0.10	−0.27^**^	−0.19^*^	−0.13	0.01	0.04	1.00

* *p < 0.05*,

** *p < 0.01 (two-tailed)*.

### Testing the Mediator Models

#### Preliminary Analyses

**Figure 3:**
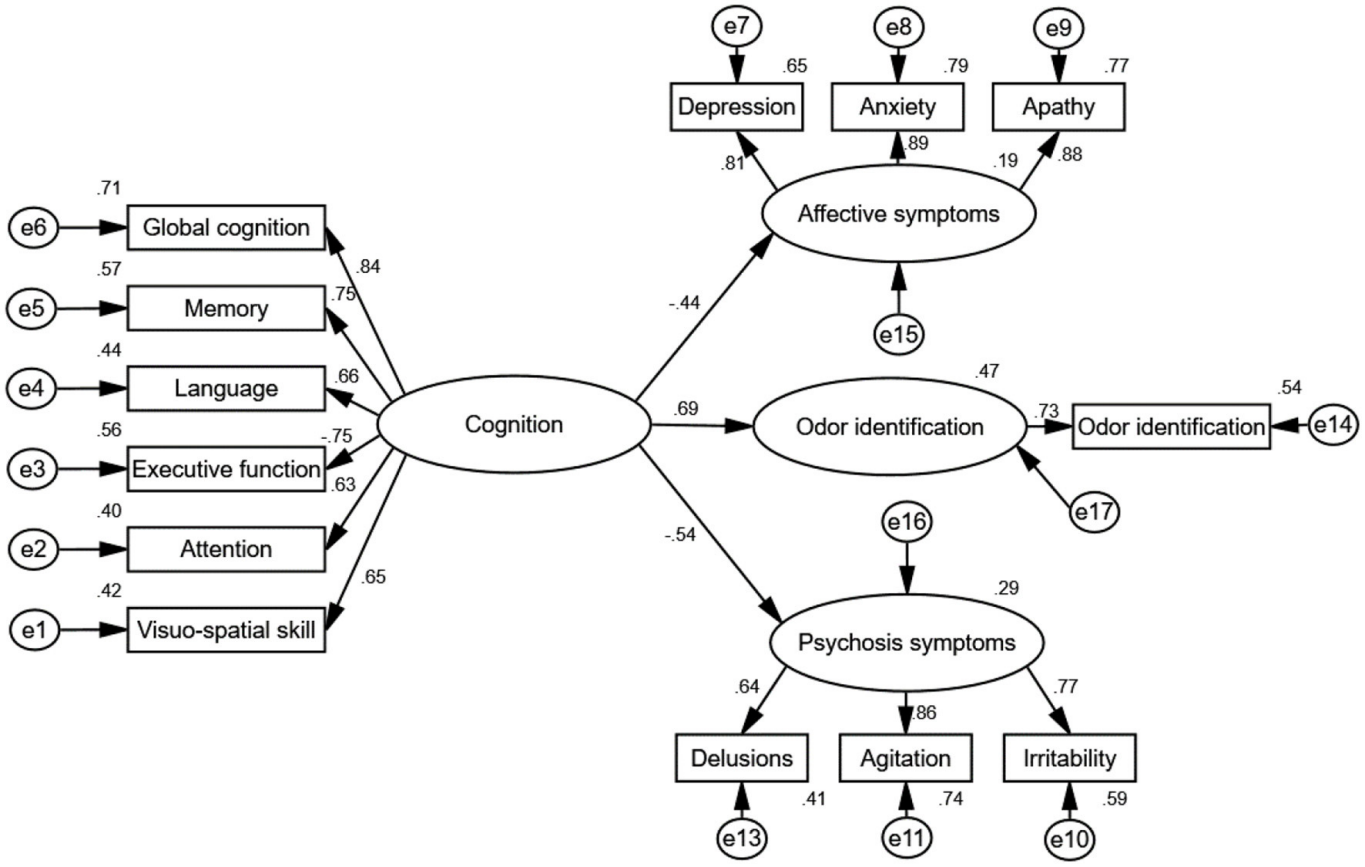
Standardized direct effects of cognition on odor identification, affective symptoms and psychosis symptoms. The goodness-of-fit indices of the model were χ^2^/*df* = 1.73, GFI = 0.91, CFI = 0.96, NFI = 0.90, IFI = 0.96, and RMSEA = 0.07, indicating a good fit. The direct effect of cognition on odor identification (standardized direct effect 0.69, *p* < 0.01), direct effect of cognition on affective symptoms (standardized direct effect 0.44, *p* < 0.01), and direct effect of cognition on psychosis symptoms (standardized direct effect −0.54, *p* < 0.01). All results indicate that cognition is associated with odor identification, affective symptoms and psychosis symptoms. e1–17 represent residuals of the respective variables.

To measure the internal consistency reliability, convergent validity, and discriminant validity of the constructs in our proposed model, we performed a CFA analysis on the four constructs of cognitive decline, affective symptoms, psychosis symptoms, and odor identification dysfunction (Figures 2, 3). The results reveal that the composite reliability (CR) of each construct ranged from 0.64 to 0.89, exceeding the 0.60 CR threshold value and giving evidence of internal consistency reliability (Fornell and Larcker, 1981; Bagozzi and Yi, 1989). In addition, the factor loadings of the individual items in the model were significant (all *p* < 0.01) (Table 4), showing preliminary evidence for the convergent validity of the measurement model. Meanwhile, the average variance extracted (AVE) values of all constructs ranged from 0.51 to 0.73, exceeding the 0.50 AVE threshold value (Fornell and Larcker, 1981; Bagozzi and Yi, 1989), denoting acceptable convergent validity.

**Table 4:** Unstandardized regression weights of the measurement model for the NPS group (*N* = 168).

**Relationship**	**Estimate**	**Standard error**	**Credit report**	***p***
Cognition → Visual-spatial skill	1.00			
Cognition → Attention	1.49	0.21	7.18	<0.01^**^
Cognition → Executive function	−6.70	0.81	−8.22	<0.01^**^
Cognition → Language	0.59	0.08	7.45	<0.01^**^
Cognition → Memory	1.30	0.16	8.25	<0.01^**^
Cognition → Global cognition	1.04	0.12	9.00	<0.01^**^
Affective symptoms → Depression	1.00			
Affective symptoms → Anxiety	1.11	0.09	12.88	<0.01^**^
Affective symptoms → Apathy	1.10	0.09	12.67	<0.01^**^
Psychosis symptoms → Agitation	1.00			
Psychosis symptoms → Irritability	0.85	0.09	9.16	<0.01^**^
Psychosis symptoms → Disinhibition	1.06	0.14	7.76	<0.01^**^

** *p < 0.01 (two-tailed)*.

#### Structural Model

We followed Baron and Kenny (1986) suggestion and used a strategy to examine the first condition of mediation. As shown in Table 3, the correlation coefficients indicate that cognition was positively associated with odor identification. In addition, the result for the direct effect of cognition on odor identification is statistically significant (standardized direct effect 0.69, *p* < 0.01, see Figure 3).

**Figure 4:**
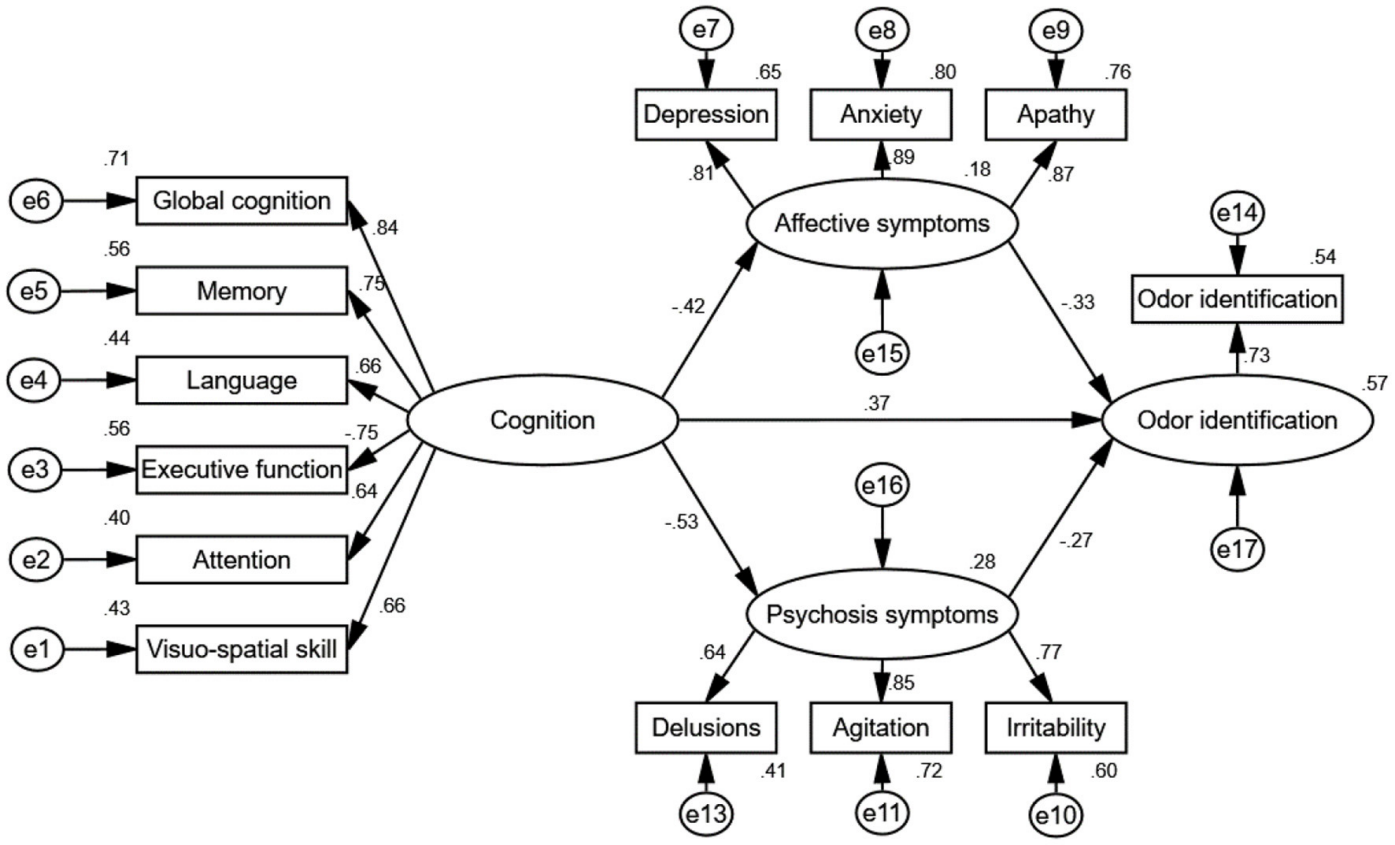
Path diagram of the standardized estimate mediation model. The goodness-of-fit indices of the mediation model were χ^2^/*df* = 1.14, GFI = 0.99, CFI = 0.99, NFI = 0.94, IFI = 0.99, and RMSEA = 0.03, indicating a good fit. The mediation model shows that cognition had special indirect effects on odor identification through affective and psychosis symptoms, indicating that affective and psychosis symptoms exhibited partial mediated effects. Rotation converged in 9 iterations. e1–17 represent residuals of the respective variables.

From test of the second condition of mediation, the results for the direct effects of cognition on affective symptoms (standardized direct effect −0.42, *p* < 0.01), the direct effect of affective symptoms on odor identification (standardized direct effect −0.33, *p* < 0.01), the direct effect of cognition on psychosis symptoms (standardized direct effect −0.53, *p* < 0.01), and the direct effect of psychosis symptoms on odor identification (standardized direct effect −0.27, *p* < 0.01) are statistically significant (Table 5; Figure 4). Therefore, the second conditions of mediation in our proposed model are supported. To investigate the special indirect effects of the dependent variable through the mediators, we performed percentile bootstrapping and bias-corrected percentile bootstrapping at a 95% confidence interval with 10,000 bootstrap samples (Taylor et al., 2008). We followed the suggestions of Hayes (2009) and calculated the confidence interval of the lower and upper bounds to test whether the special indirect effects were significant. As shown in Table 5, the results of the bootstrap test confirm the existence of positive and significant special indirect effects for affective and psychosis symptoms between cognition and odor identification. However, we found no difference in special indirect effects between affective and psychosis symptoms. Thus, affective and psychosis symptoms partially mediated the effect of cognition on odor identification.

**Table 5:** Unstandardized total, direct, indirect, and specific indirect effects of the mediation model (*N* = 168).

**Relationship**	**Point estimate**	**Product of coefficient**		**Bootstrapping**					
				**Bias-corrected**			**95% percentile**		
		***SE***	***Z***	**Lower**	**Upper**	**p**	**Lower**	**Upper**	***p***
**Total effects**									
Cognition → Affective symptoms	−0.28	0.08	−3.71	−0.47	−0.16	<0.01^**^	−0.45	−0.15	<0.01^**^
Cognition → Psychosis symptoms	−0.22	0.06	−3.91	−0.34	−0.12	<0.01^**^	−0.33	−0.11	<0.01^**^
Cognition → Odor identification	0.33	0.07	4.87	0.21	0.47	<0.01^**^	0.21	0.47	<0.01^**^
Affective symptoms → Odor identification	−0.25	0.08	−3.10	−0.41	−0.10	<0.01^**^	−0.39	−0.08	<0.01^**^
Psychosis symptoms → Odor identification	−0.34	0.19	−1.82	−0.72	−0.02	<0.01^**^	−0.69	−0.01	<0.01^**^
**Direct effects**									
Cognition → Affective symptoms	−0.28	0.08	−3.71	−0.47	−0.16	<0.01^**^	−0.45	−0.15	<0.01^**^
Cognition → Psychosis symptoms	−0.22	0.06	−3.91	−0.34	−0.12	<0.01^**^	−0.33	−0.11	<0.01^**^
Cognition → Odor identification	0.19	0.08	2.47	0.04	0.35	0.01^*^	0.04	0.36	0.01^*^
Affective symptoms → Odor identification	−0.25	0.08	−3.10	−0.41	−0.10	<0.01^**^	−0.39	−0.08	0.01^*^
Psychosis symptoms → Odor identification	−0.34	0.19	−1.82	−0.72	−0.02	0.03^*^	−0.69	−0.01	0.04^*^
**Indirect effects**									
Cognition → Odor identification	0.14	0.05	2.71	0.07	0.28	<0.01^**^	0.05	0.26	<0.01^**^
**Special indirect effects**									
Cognition → Affective symptoms → Odor identification	0.07	0.03	2.38	0.03	0.14	<0.01^**^	0.02	0.13	0.01^*^
Cognition → Psychosis symptoms → Odor identification	0.07	0.04	1.64	0.01	0.20	0.02^*^	0.00	0.17	0.04^*^
Different of Special indirect effects	0.00	0.05	0.06	−0.10	0.11	0.94	−0.10	0.11	0.94

*Standardized estimation of 10,000 bootstrap samples. SE, standard error. p, two-tailed significance*.

* < 0.05, ** < 0.01.

## Discussion

The present study explored the relationship between odor identification, NPS and cognition in patients with AD and MCI via SEM. The main findings of this study are as follows: (1) patients with NPS exhibited significantly poorer odor identification and cognition scores than those without NPS and HCs. (2) In patients with NPS, odor identification scores were negatively correlated with affective and psychosis symptoms (factors extracted from the NPI-12) but not with behavioral symptoms. (3) Affective and psychosis symptoms exhibited specific indirect effects between odor identification and cognition. Moreover, there were positively mediated effects of affective and psychosis symptoms on the relationship between odor identification and cognition.

The present SEM analyses suggest additive effects of NPS and odor identification dysfunction on cognitive decline where affective and psychosis symptoms partially mediate the relationship between odor identification dysfunction and cognitive decline. Previous studies demonstrate that both odor identification dysfunction and NPS are common in patients with AD and MCI and that odor identification dysfunction could serve as a marker of indicating early AD pathology and cognitive decline (Devanand et al., 2015; Lafaille-Magnan et al., 2017; Murphy, 2019; Zhao et al., 2020; Wang et al., 2021), but few consider the potentially confounding effect of NPS.

The current study shows that among the three factors of NPS, affective symptoms are most related to odor identification and cognitive decline, and they show a partial mediation of the relationship between cognitive decline and odor identification. It is well acknowledged that numerous brain areas that are altered with affective symptoms and cognitive impairment are also involved in olfaction processing, such as that occurring in the olfactory bulb, hippocampus, amygdala, insula, orbitofrontal cortex, and habenula nucleus (Li et al., 2015; Croy and Hummel, 2017). For depression, Croy et al. identified potential mechanisms linking depression and olfactory dysfunction. First, decreased olfactory function in depression may result from decreased attention to olfaction and a consecutively decreased turnover rate of olfactory receptor neurons in the olfactory epithelium where these effects are temporary and diminish after remission (Li et al., 2015). Second, a smaller olfactory bulb volume may cause decreased signaling from the olfactory bulb to the amygdala, hippocampus, striatum and orbitofrontal cortex (Croy and Hummel, 2017). Similar to what is observed in depression, there are direct connections between olfactory relay neurons and the amygdala, a key node in the regulation of anxiety (Ballanger et al., 2019). Moreover, depression and anxiety can also lead to decreased neurogenesis in the hippocampus and olfactory bulb (Mineur et al., 2007), and olfactory training seems to increase olfactory bulb volume (Negoias et al., 2016), cognitive function (Birte-Antina et al., 2018), and depressive and anxious symptoms (Ballanger et al., 2019). For apathy, a previous study reported a specific association between odor identification dysfunction and the severity of apathy, suggesting that olfactory dysfunction and apathy might result from the progression of disease pathology in shared neural substrates (Seligman et al., 2013). Previous studies have already shown that depression, apathy, and anxiety are precursor symptoms and can predict cognitive decline in the AD spectrum (Craig et al., 2005; Seligman et al., 2013; Ma, 2020). Given the close relationships between odor identification dysfunction, affective symptoms, and cognitive decline, the confounding effect of affective symptoms should be adjusted when using odor identification dysfunction to predict cognitive decline in patients with AD and MCI.

Similar to affective symptoms, psychosis symptoms also had a partial mediated effect on the relationship between cognitive function and odor identification. Previous research indicates that odor identification dysfunction is present in schizophrenia patients (Moberg et al., 2006), first-degree relatives of schizophrenia patients (Turetsky et al., 2008), and subjects at risk for psychosis symptoms (Takahashi et al., 2018; Tang et al., 2018) and is believed to be highly associated with disease duration (Moberg et al., 2006), negative symptoms, and social-cognitive function (Corcoran et al., 2005). The anatomic proximity of the olfactory network to limbic structures provides a potential explanation for the relation between olfaction and affective symptoms, and may account for their shared dysfunction in various psychiatric disorders. Many of these regions, including the amygdala, hippocampus, insula, anterior cingulate cortex, and orbitofrontal cortex, have been described as common neural substrates for emotional symptoms, psychosis symptoms, and olfactory processing. Similarly, psychosis symptoms were also found to be strongly associated with cognitive decline in AD and MCI (Mallo et al., 2020). Overall, because psychosis symptoms heavily interact with abnormalities of olfactory and cognitive processing, their confounding effect should be considered when exploring the relationship between odor identification dysfunction and cognitive decline in patients with AD and MCI.

No significant association was observed between behavioral symptoms and odor identification, indicating that odor identification dysfunction may be independent of the presence of behavioral symptoms. Previous studies have suggested that affective symptoms are commonly observed from early stages of the disease (Craig et al., 2005) and that psychosis symptoms are more obvious at a more advanced AD stage (Piccininni et al., 2005), while behavioral symptoms are more often considered to be a transient symptom that fluctuates throughout disease progression (Garre-Olmo et al., 2010). On the other hand, behavioral symptoms are not linked to specific brain regions, and studies have suggested that they may be linked to dysfunctions in the posterior cingulate cortex, frontal cortex, and bilateral parietal lobes (Liu et al., 2004; Ng et al., 2021) which are less associated with olfactory processing. Thus, the effect of behavioral symptoms on cognitive decline and odor identification dysfunction is less significant or even undetectable compared to affective and psychosis symptoms.

The present study has limitations. (1) The results are based on cross-sectional analyses, and the causal relationships between olfactory dysfunction, NPS, and cognitive decline must be further explored in longitudinal studies. (2) The present study demonstrates that NPS mediated the relationship between cognitive decline and odor identification dysfunction, but whether they mediated the relationship between odor identification dysfunction and neurodegeneration needs to be confirmed by future studies involving, for example, the assessment of cerebrospinal fluid biomarkers and PET-CT. (3) The current study only involved an assessment of odor identification, because it is the strongest predictor of AD. However, significant associations between NPS and other aspects of olfaction (such as odor thresholds and odor discrimination) have also been reported in previous studies (Moberg et al., 2006; Pollatos et al., 2007; Chen et al., 2019). Future studies including odor thresholds and discrimination could provide a deeper understanding of how olfaction and NPS interact with each other in patients with AD/MCI. (4) Reduced self-awareness is associated with some NPS (disinhibition, apathy, anxiety, executive dysfunctions) in AD spectrum and a more aggressive progression of MCI and AD (Amanzio et al., 2011, 2020). Therefore, to minimize the effect of awareness, the present study used objective assessments rather than self-report scales for measuring odor identification, cognitive function, and NPS. However, we are not sure whether the awareness may mildly affect the result.

## Conclusion

In summary, the current study demonstrates that NPS mediate the relationship between odor identification dysfunction and cognitive decline in patients with MCI and AD. When odor identification is used to predict cognitive decline in patients with AD and MCI, the confounding effect of affective symptoms and psychosis symptoms should be taken into account. Longitudinal studies must explore the causal relationships between olfactory dysfunction, NPS, and cognitive decline, and neuroimaging and CSF markers could better clarify the underlying mechanisms through which olfactory dysfunction, NPS, and cognitive decline interact with each other.

## Data Availability Statement

The raw data supporting the conclusions of this article will be made available by the authors, without undue reservation, to any qualified researcher.

## Ethics Statement

The studies involving human participants were reviewed and approved by the Ethics Committees of the Affiliated Brain Hospital of Guangzhou Medical University. The patients/participants provided their written informed consent to participate in this study. Written informed consent was obtained from the individual(s) for the publication of any potentially identifiable images or data included in this article.

## Author Contributions

All authors contributed to the writing and revision of the manuscript, read, and approved the final manuscript.

## Funding

This study was supported by a grant from the National Natural Science Foundation of China (No. 81701341); Guangzhou Municipal Psychiatric Diseases Clinical Transformation Laboratory (No. 201805010009); Key Laboratory for Innovation Platform Plan, the Science and Technology Program of Guangzhou, China, Science and Technology Plan Project of Guangdong Province (No. 2019B030316001); and National Key Research and Development Program of China (No. 2016YFC0906300). The funders had no role in the study design, data collection and analysis, decision to publish or preparation of the manuscript.

## Conflict of Interest

The authors declare that the research was conducted in the absence of any commercial or financial relationships that could be construed as a potential conflict of interest.

## Publisher's Note

All claims expressed in this article are solely those of the authors and do not necessarily represent those of their affiliated organizations, or those of the publisher, the editors and the reviewers. Any product that may be evaluated in this article, or claim that may be made by its manufacturer, is not guaranteed or endorsed by the publisher.

## Abbreviations

Ab. Mot. Beh: aberrant motor behavior
AD: Alzheimer's disease
AVE: average variance extracted
AVLT: auditory verbal learning test
BNT: Boston naming test
CFA: confirmatory factor analysis
CFI: comparative fix index
CR: composite reliability
EFA: exploratory factor analysis
HCs: healthy controls
IFI: Bollen's incremental fit index
KMO: Kaiser–Meyer–Olkin
MCI: mild cognitive impairment
MMSE: mini-mental state examination
NFI: normed fit index
No-NPS: no neuropsychiatric symptoms
NPI: neuropsychiatric Inventory
NPS: neuropsychiatric symptoms
RMSEA: root mean square error of approximation
ROCF: ReyO-Sterrieth complex figure test
SDMT: symbol-digit modality test
SE: standard error
SEM: structural equation modeling
SRMR: standardized root mean square residual
TMT: trail-making test.
